# Supplementary material for: Mechanism-based and computational modeling of hydrogen sulfide biogenesis inhibition: interfacial inhibition
Source: Sci Rep. 2023 May 4;13:7287. doi: 10.1038/s41598-023-34405-3 (PMC10160035; doi:10.1038/s41598-023-34405-3)
Supplement: Supplementary file 1 — Supplementary Information. [file 41598_2023_34405_MOESM1_ESM.pdf]

Supporting Information

Mechanism-Based and Computational Modeling of Hydrogen Sulfide Biogenesis Inhibition - Interfacial Inhibition

Laurent Le Corre and Dominique Padovani*

Université Paris Cité, CNRS, Laboratoire de Chimie et de Biochimie Pharmacologiques et Toxicologiques, F-75006 Paris, France.

*Corresponding author. Email: dominique.padovani@parisdescartes.fr

Contents

Table S1	Effect of D-pen on the distances substrate-O3' or -C4' and on the perimeter of the substrate access channel entrance recorded after 50 ns MD simulations..	S1
Figure S1	Purification of recombinant human cystathionine γ-lyase.....................	S2
Figure S2	Influence of the N-terminal 6xHis-tag on CSE activity ………………….	S3
Figure S3	Mechanism and characteristics of mixed inhibition……………………..	S4
Figure S4	Inhibition of H_2S or Cys production by D-penicillamine………………….	S5
Figure S5	RMSD and RMSF plots…………………………………………………….	S6
Figure S6	Interacting networks of PLP and cystathionine from molecular docking……………………………………………………………………….	S8
Figure S7	Structural comparison of the impact of D-pen binding to CSE on the interacting network of PLP after 50 ns MD simulations…………………	S9
Figure S8	Structural comparison of the impact of D-pen binding to CSE on the interacting network of cystathionine after 50 ns MD simulations………	S10
Figure S9	Two-dimensional substrate- and ligand-protein interaction diagrams obtained after 50 ns MD simulations	S11
Figure S10	Implication of the O3' atom from PLP into proton transfer....................	S12
Figure S11	Representative docking pose for the binding of D-pen at site 42…….	S13
Figure S12	Structural comparison of D-penicillamine binding at site 12 after docking and MD simulations………………………………………………	S14
Figure S13	Structural comparison of D-penicillamine at site 33 after docking and MD simulations…………………………………………………………..	S15

Table S1 - Effect of D-pen on the distances d(N_{CST},O3'$_{PLP}$), d(N_{CST},C4'$_{PLP}$) and on the perimeter* of the substrate access channel entrance (in Å) recorded after 50 ns MD simulations.

	d(N_{CST},O3'$_{PLP}$)	d(N_{CST},C4'$_{PLP}$)	Perimeter
**No D-Pen**	2.86	3.62	33.6
**D-Pen at site 12**	5.80	4.88	37.1
**D-Pen at site 33**	6.26	6.05	48.8

*The perimeter recorded before MD is of 38.0 Å

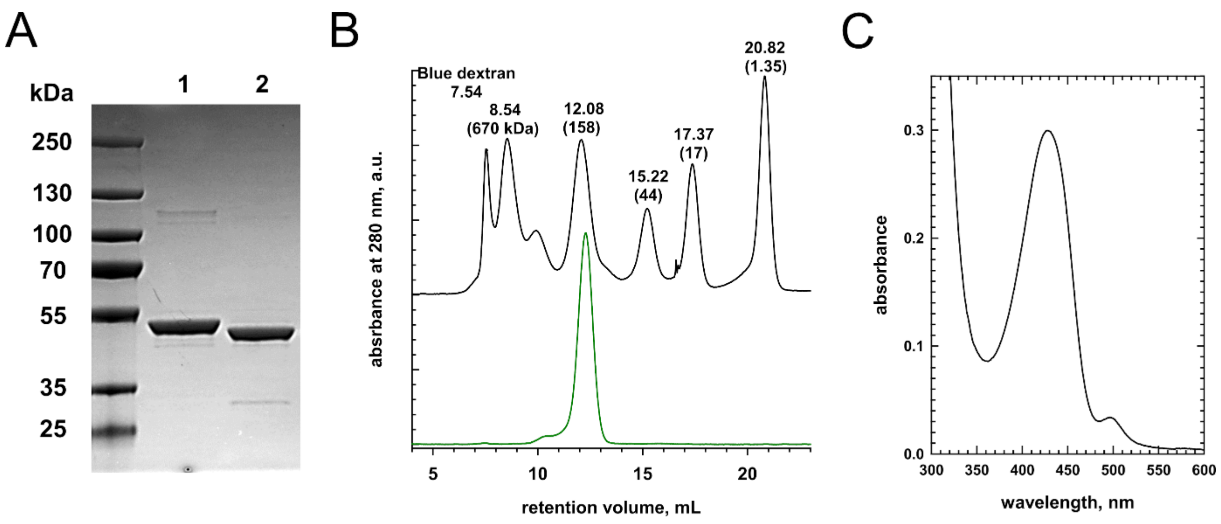

Figure S1 - Purification of recombinant human cystathionine γ-lyase. (**A**) 12% SDS-polyacrylamide gel of recombinant human CSE (12 μg) before (**1**) and after (**2**) cleavage of its $N_{terminal}$ 6xHis-tag by TEV protease. The molecular masses of the Precision Plus Protein standards (left lane, Bio-Rad) are shown. (**B**) Gel filtration analysis of purified recombinant tag-less human CSE (100 μg - green line) on a 10/300 GL Superdex-200 size exclusion column (GE Healthcare) eluted with 100 mM HEPES, 150 mM NaCl at pH 7.4 at 0.5 mL/min. The elution profile of (Bio-Rad) protein standards (black line) separated under the same conditions is shown for comparison. The apparent retention volume and molecular weights of protein standards used are labelled on the plot. The latter are thyroglobulin (670 kDa), bovine γ-globulin (158 kDa), chicken ovalbumin (44 kDa), equine myoglobin (17 kDa), and vitamin B_{12} (1.35 kDa). The retention volume of tag-less human CSE (12.220 ± 0.062 mL, n=2±SD) corresponds to an apparent molecular weight of 148 ± 4 kDa. Under similar conditions, full-length CSE eluted with a retention volume of 12.157 ± 0.045 mL (n=2±SD), which corresponds to an apparent molecular weight of 152 ± 3 kDa. (**C**) UV-Visible spectrum of the PLP cofactor of purified recombinant human cystathionine γ-lyase monitored in 100 mM HEPES at pH 7.4 and 20°C. The internal aldimine form of the PLP cofactor exhibits a sharp band at 428 nm. A small proportion of purified CSE contains PLP-aminoacrylate species that displays an absorption peak around 495 nm.

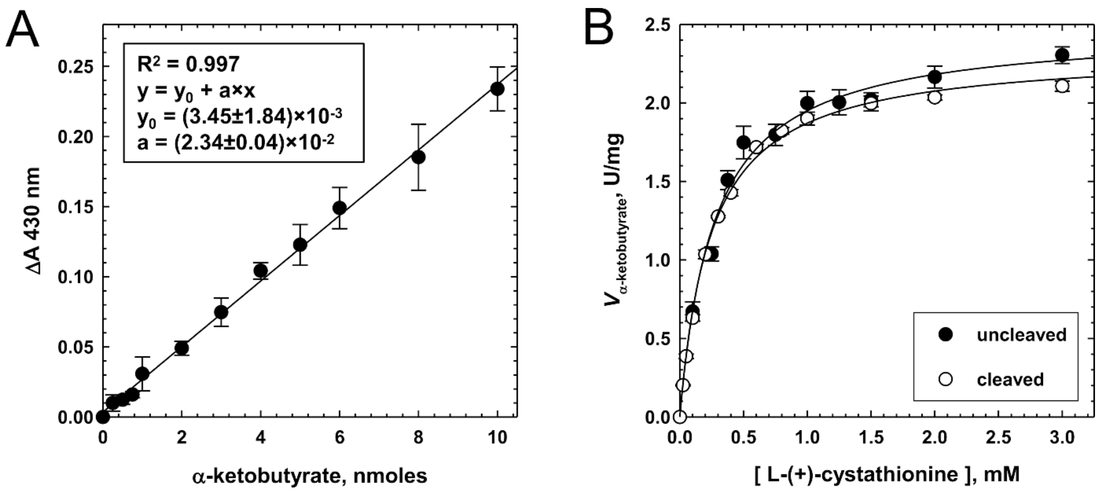

Figure S2 - Influence of the N-terminal 6xHis-tag on CSE activity. (**A**) Etalon curve for the determination of α-ketobutyrate content in the 2,4-dinitrophenyl hydrazine test (n = 4 ± SD). (**B**) Kinetics of α-ketobutyrate generation from cystathionine by CSE (uncleaved) and 6xHis-tag-less CSE (cleaved) (n = 2 ± SD). Cysteine synthesis from L-(+)-cystathionine was determined by measuring α-ketobutyrate production at 37°C from assay mixtures containing 0.1-4 mM L-(+)-cystathionine and CSE (10 μg) in 100 mM HEPES at pH 7.4. Hyperbolic fit to the data gave V_{max} and K_M values of 2.47 ± 0.05 U/mg and 0.269 ± 0.020 mM for CSE, and 2.33 ± 0.02 U/mg and 0.246 ± 0.006 mM for tag-less CSE.

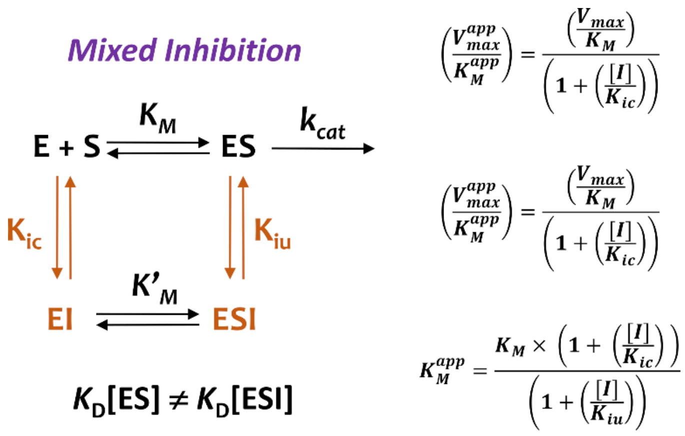

Figure S3 - Mechanism and characteristics of mixed inhibition. E, enzyme; S, substrate; ES, Michaelis complex; I, inhibitor.

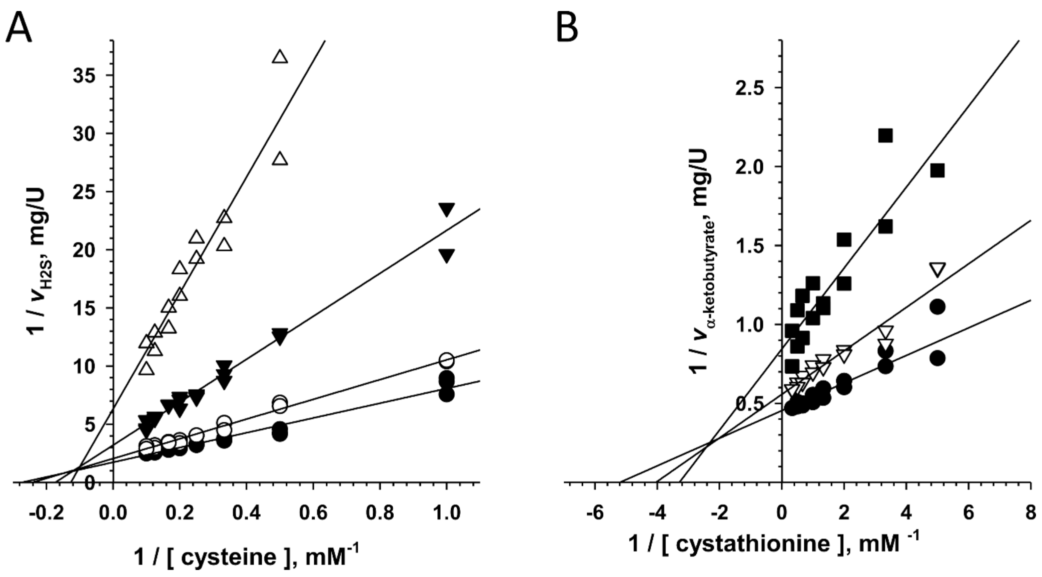

Figure S4 - Inhibition of H_2S production and cystathionine cleavage by D-penicillamine. (**A**) Lineweaver-Burk plot analysis of CSE inhibition by D-penicillamine (n ≥ 2 ± SD) as determined in **Fig.3B**. Assay mixtures contained 1-10 mM cysteine, 0.4 mM lead acetate, CSE (40 μg) and varying concentrations of D-pen (●, 0 mM; ○, 1 mM; ▼, 5 mM; △, 10 mM) in 100 mM HEPES at pH 7.4 and 37 °C. (**B**) Lineweaver-Burk plot analysis of CSE inhibition by D-penicillamine (n = 2 ± SD) as determined in **Fig.4A**. Assay mixtures contained 0.1-3 mM cystathionine, CSE (5 μg) and varying concentrations of D-pen (●, 0 mM; ▽, 2 mM; ■, 5 mM) in 100 mM HEPES at pH 7.4 and 37 °C.

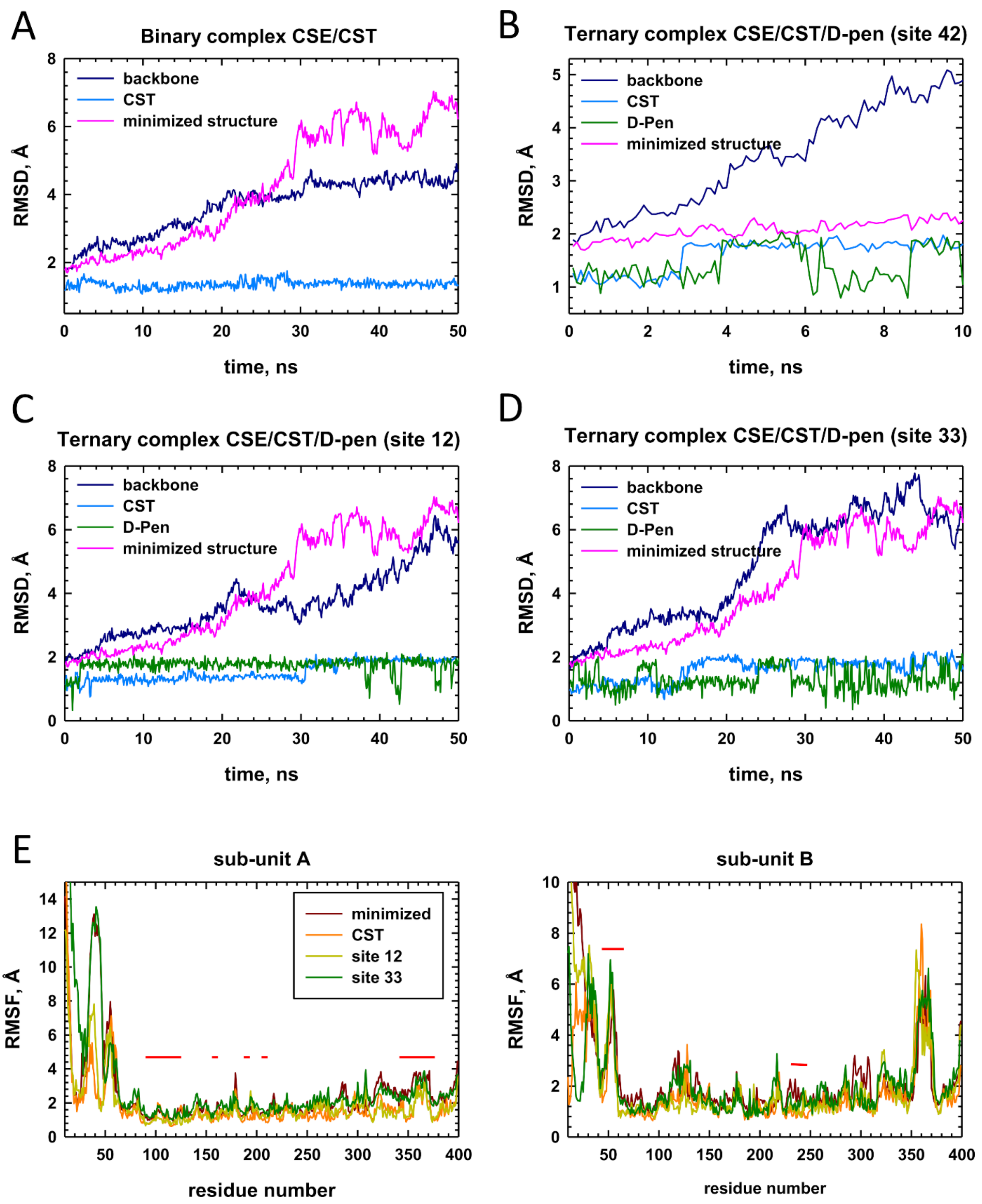

Figure S5 - RMSD and RMSF plots. (**A-D**) Root mean square deviation (RMSD) analysis was carried out for the molecular dynamics simulations of each system (top and middle plots). The RMSD analysis of the minimized structure is shown in each plot for comparison. (**E**) The RMSF (for the backbone of each residue) of minimized, binary and ternary complexes trajectories were calculated along the entire sampling interval (50 ns). The horizontal red lines indicate the position of amino acids that are integral to the active site and participate in the substrate channel unity. An analysis of the RMSD of CST and D-pen show that substrate and ligand are

both stable in their respective binding pocket along the entire dynamics range (50 ns). Importantly, only active site residues belonging to the flexible loop[44-65] from the B subunit (structural element I - see Fig. 5*A*) seem to display some flexibility during molecular dynamics simulations. Reduced RMSD value of the enzyme and reduced RMSF of the enzyme residues observed for the binary CSE-CST complex indicate that substrate binding stabilizes the complex.

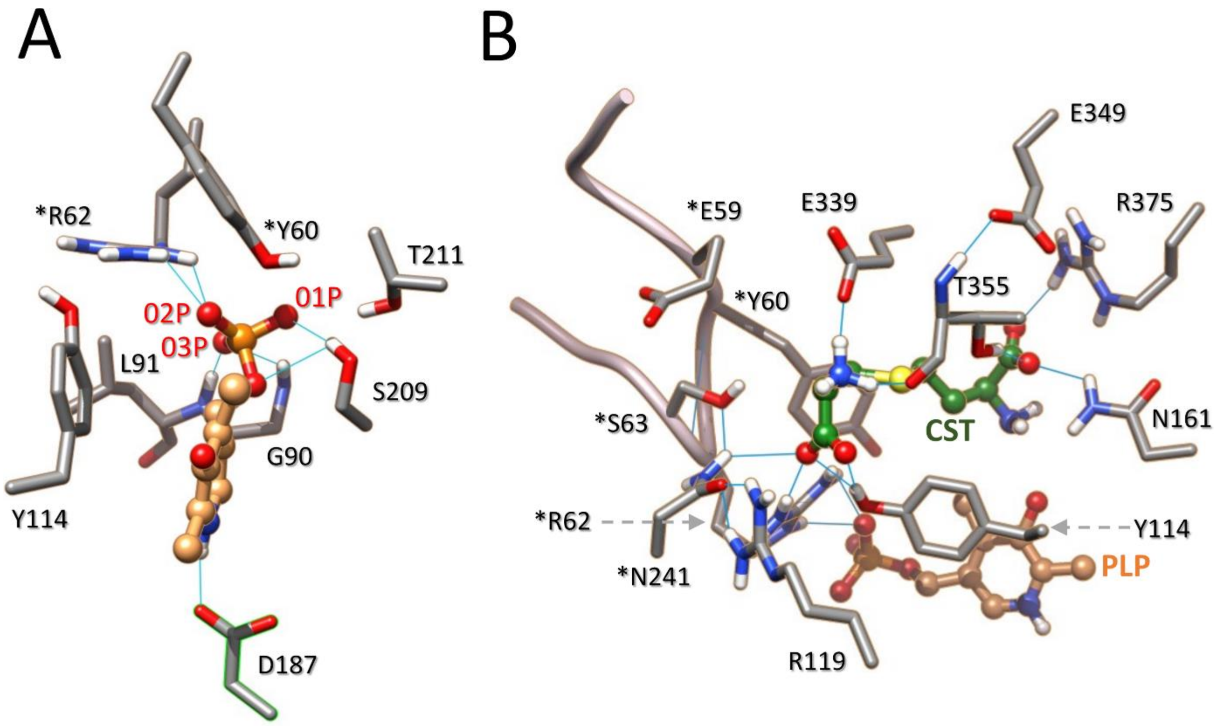

Figure S6 - Interacting networks of PLP and cystathionine from molecular docking. Representative docking poses showing the interacting network of PLP (**A**) and cystathionine (**B**) in the active site from the A subunit. The imine bond between the C4’ atom of PLP and Lys212 has been omitted for clarity. *Residue from the adjacent B subunit. H-bonds are depicted by light blue lines.

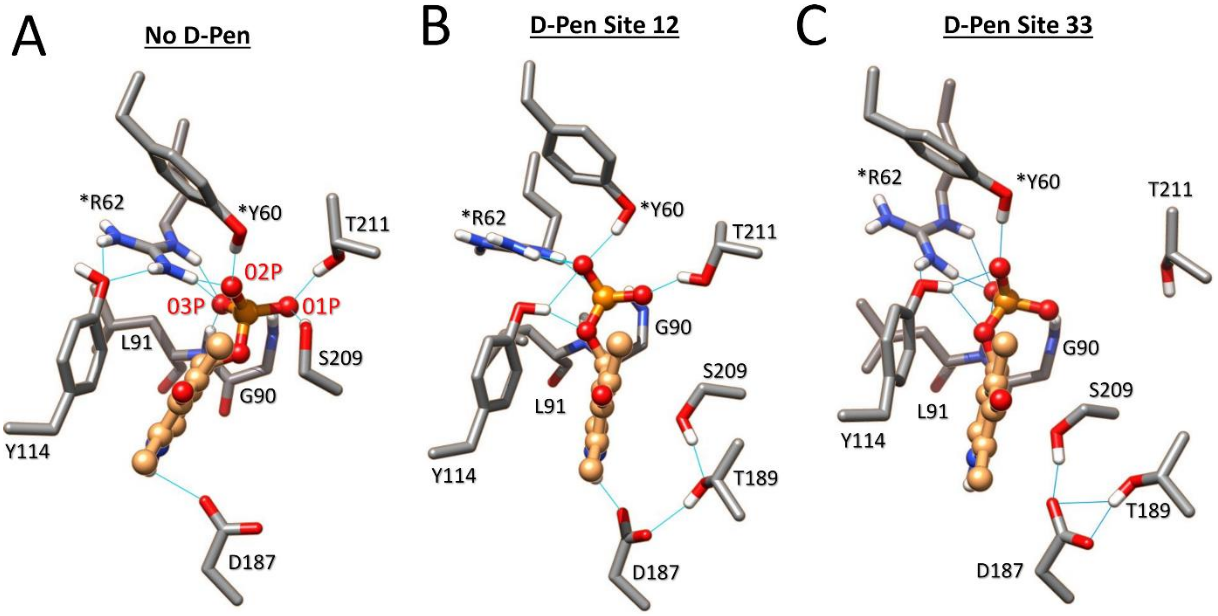

Figure S7 - Structural comparison of the impact of D-pen binding to CSE on the interacting network of PLP after 50 ns MD simulations. Structural comparison of the H-bond network of PLP in the active site from the A subunit during MD at t=50 ns in the absence (**A**) or the presence of D-penicillamine bound to site 12 (**B**) or 33 (**C**). Significantly, the PLP cofactor is destabilized at its phosphate group and/or its pyridine moiety when D-penicillamine is bound to site 12 or site 33. Thus, the phosphate group of the cofactor no longer establishes H bond contacts with the backbone of Gly90 and Leu91 when D-pen is bound to site 12 while the PLP is no more stabilized by H-bonding with Thr211 and no more activated by N-protonation in the presence of D-Pen at site 33. In addition, Tyr114 falls within a radius of hydrogen interaction with O2P and O4P atoms from PLP in the presence of the ligand. *Residues from the adjacent subunit B. H-bonding are depicted by light blue lines.

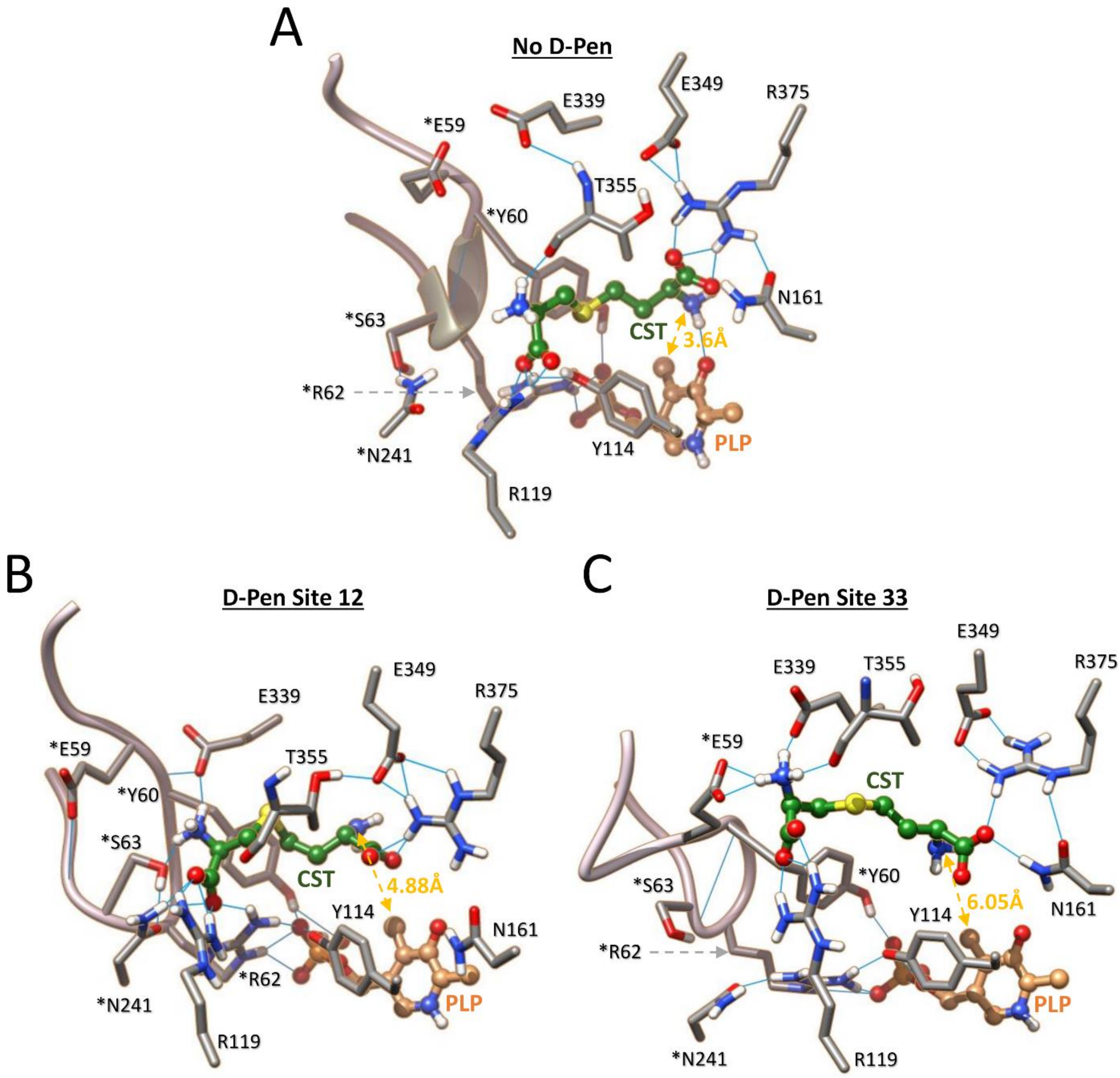

Figure S8 - Structural comparison of the impact of D-pen binding to CSE on the interacting network of cystathionine after 50 ns MD simulations. Structural comparison of the interacting network of cystathionine in the active site from the A subunit in the absence (**A**) or the presence of D-penicillamine bound to site 12 (**B**) or 33 (**C**). Importantly, the substrate establishes H bond contacts with amino acid residues located at the entrance of the substrate access channel in the presence of D-pen, *i.e.* Glu339, *Ser63 and *Asn241 (site 12) or Glu339 and *Glu59 (site 33). In addition, the interacting network of cystathionine is disrupted with the distances (in yellow) between its nucleophilic amino group and the O3' or C4' atoms of the PLP significantly increased. *Residues from the adjacent subunit B. H-bonding are depicted by light blue lines.

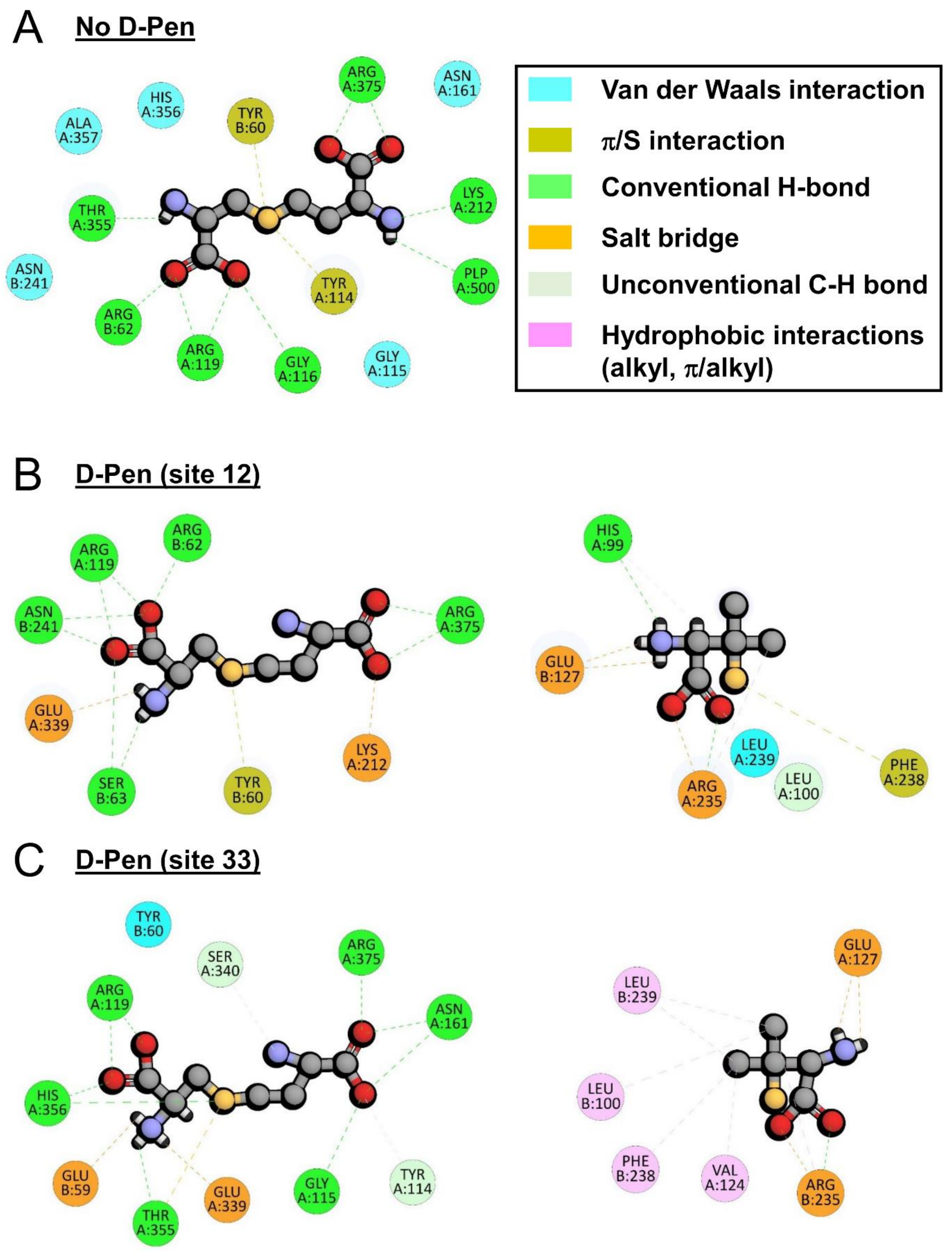

Figure S9 - Two-dimensional substrate- and ligand-protein interaction diagrams obtained after 50 ns MD simulations. Comparison of the two-dimensional cystathionine-CSE interaction diagrams in the absence (**A**) or the presence of D-penicillamine bound to site 12 (**B**, left) or 33 (**C**, left). Also shown are the two-dimensional D-Pen-CSE interaction diagrams when D-Pen is in site 12 (**B**, right) or 33 (**C**, right). The diagrams were generated with the Show 2D Diagram tool from Biovia Discovery Studio.

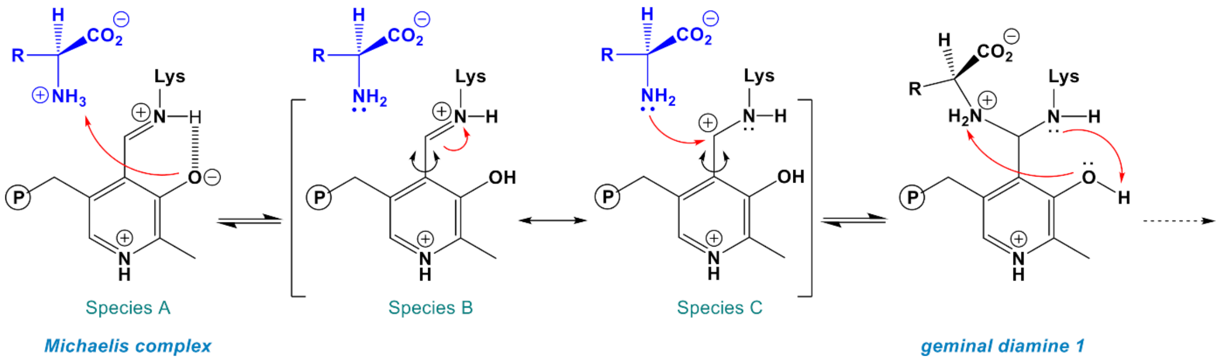

Figure S10 - Implication of the O3' atom from PLP into proton transfer. Catalytic role of O3' atom in the proton transfer from the substrate (blue) to the lysine amino group from the Schiff base linkage, as postulated for the L-serine hydratase from *Xanthomonas oryzae* pv. *Oryzae*.

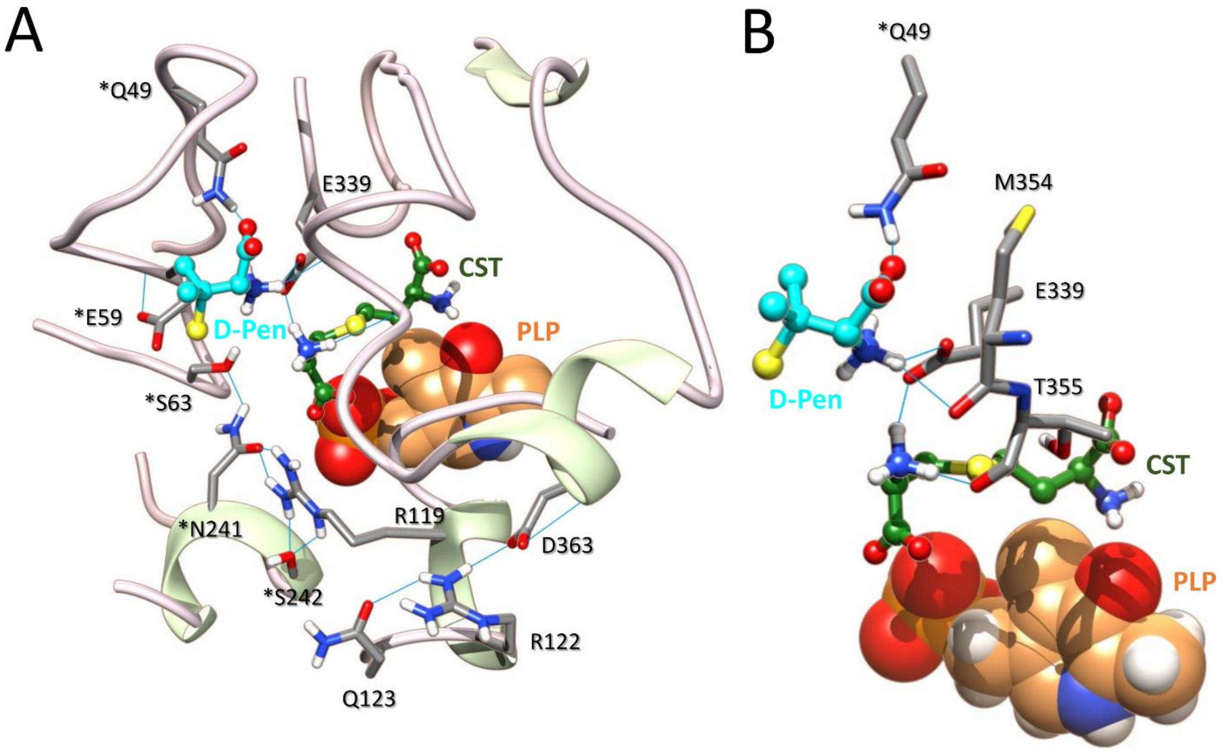

Figure S11 - Representative docking pose for the binding of D-penicillamine at the interfacial binding site 42. (**A**, **B**) Structure of a representative docking pose showing the binding location of D-pen at site 42 (**A**) and close-up of the same docking pose showing the interacting network of the ligand (**B**). As shown, D-penicillamine binds on top of the entrance of the substrate access channel from the A subunit and establishes amongst others H bond contact with Glu339 which is also implicated in substrate stabilization. *Residues from the adjacent subunit B. H-bonding are depicted by light blue lines.

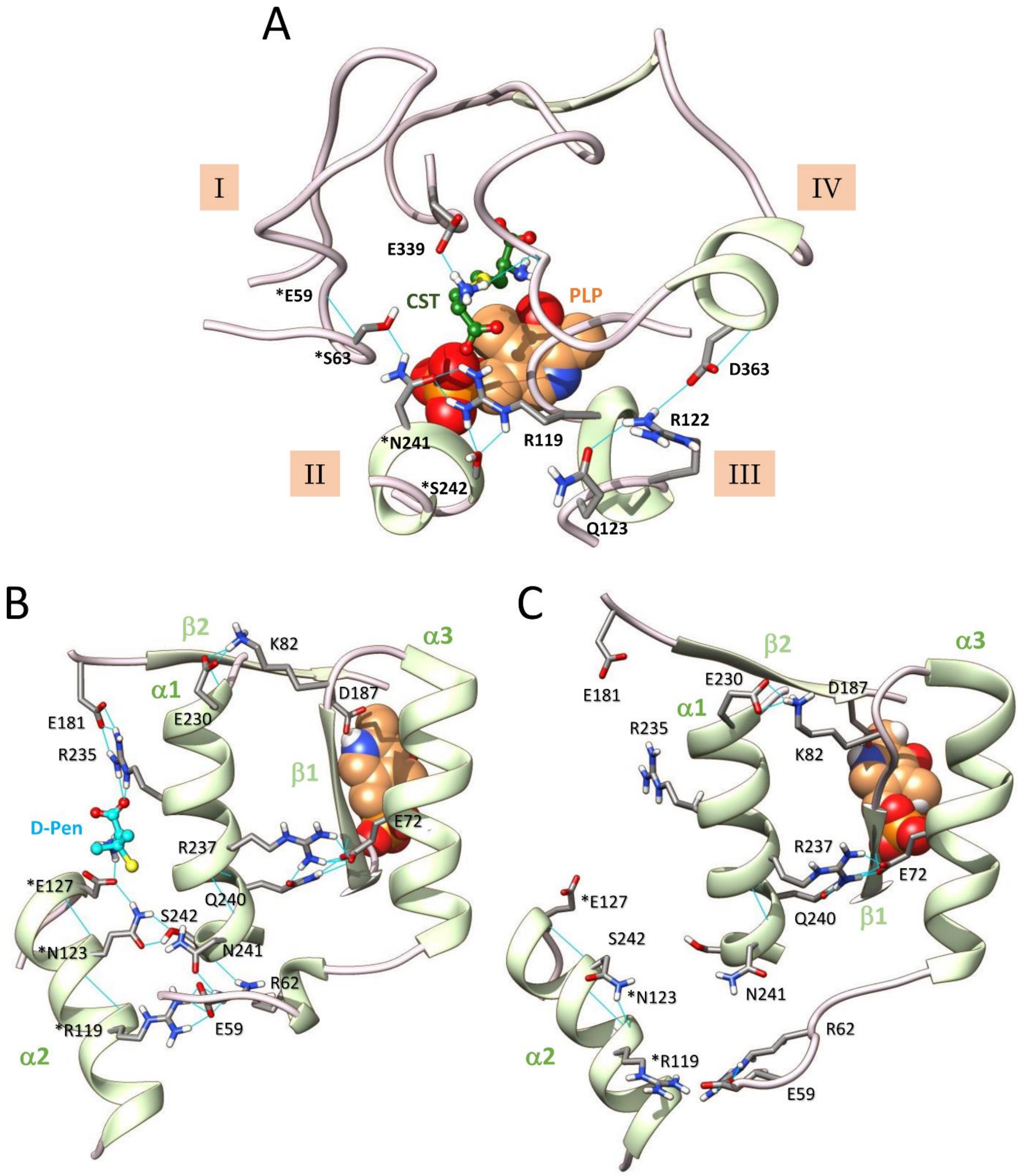

Figure S12 - Structural comparison of D-penicillamine binding at site 12 after docking and MD simulations. (**A**) Representative docking pose of the active site when D-pen is bound to site 12. (**B**, **C**) Structural comparison of representative snapshots obtained after 50 ns of molecular dynamics of the interfacial D-pen binding site 12 in the ternary (**B**) or binary (**C**) complexes. As shown, the comparison of both structures reveal that D-pen binding between interfacial α-helices α1 and α2 promotes substantial structural changes in the vicinity of the interface. * Residue from the adjacent B subunit B. H-bonds are depicted by light blue lines.

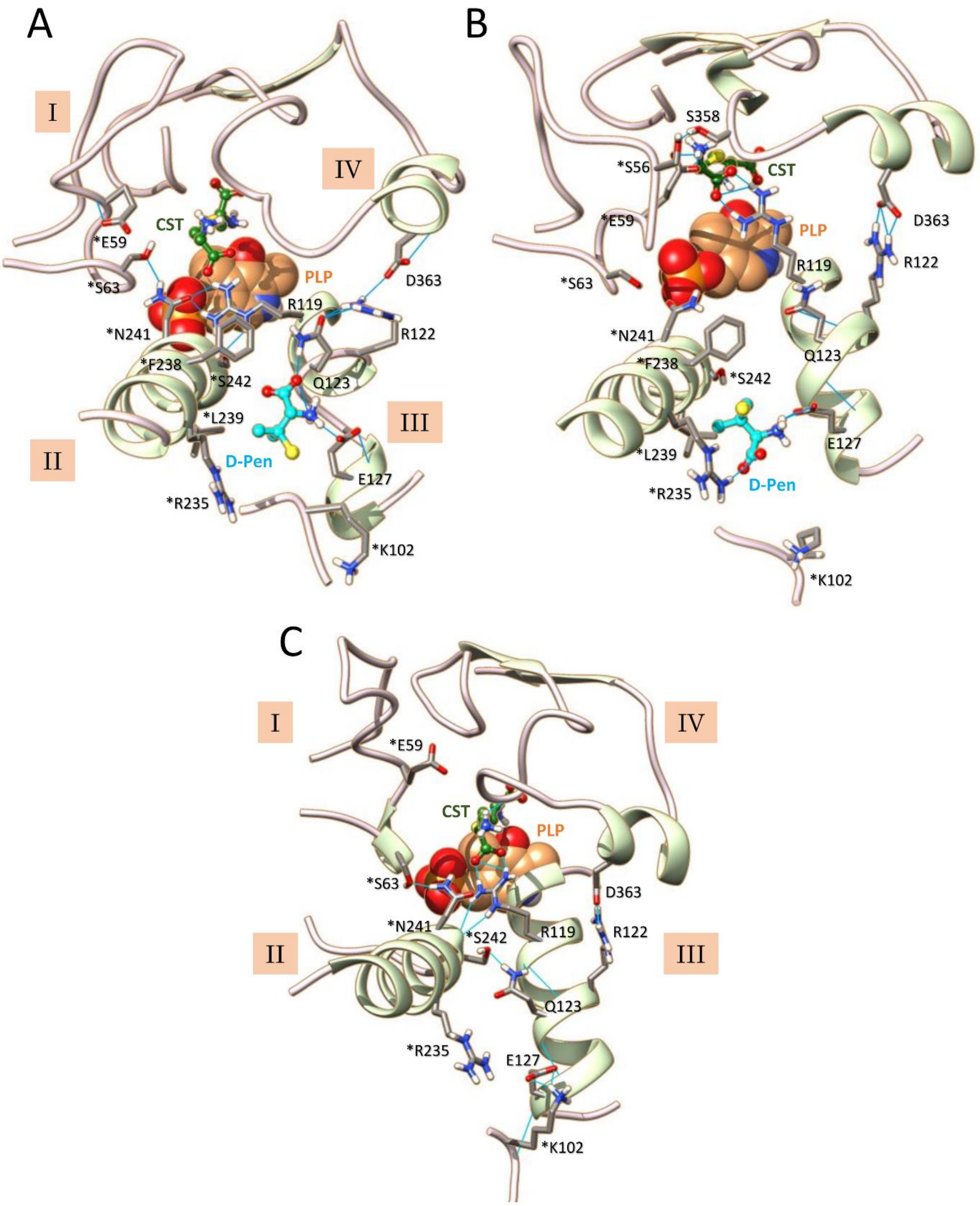

Figure S13 - Structural comparison of D-penicillamine at site 33 after docking and MD simulations. (**A**-**C**) Structural comparison of the active site from the A subunit in the presence of D-pen at site 33 after molecular docking (**A**) and 50 ns MD simulations of the ternary CSE-substrate-ligand complex (**B**) or the binary CSE-substrate complex (**C**). As shown, MD simulations induce structural changes as well as modifications of the hydrogen bonding network observed in the docking pose in the presence of D-pen. In particular, the structural elements I and IV becomes connected through a H bond between *Ser56 and Ser358 while the connection between structural elements II and III is disrupted. Importantly, the perturbations recorded in the presence of D-pen during 50 ns MD simulations are not observed

in the absence of the ligand, thus suggesting that the fixation of D-pen specifically induces structural changes in the vicinity of the active site and at the interfacial ligand binding site. *Residues from the adjacent subunit B. H-bonding are depicted by light blue lines.
